# Role of microglia in brain development after viral infection

**DOI:** 10.3389/fcell.2024.1340308

**Published:** 2024-01-16

**Authors:** Pei Xu, Yongjia Yu, Ping Wu

**Affiliations:** ^1^ Department of Neurobiology, University of Texas Medical Branch, Galveston, TX, United States; ^2^ Department of Radiation Oncology, University of Texas Medical Branch, Galveston, TX, United States

**Keywords:** microglia, innate immunity, neural progenitor cell, brain development, virus, infection

## Abstract

Microglia are immune cells in the brain that originate from the yolk sac and enter the developing brain before birth. They play critical roles in brain development by supporting neural precursor proliferation, synaptic pruning, and circuit formation. However, microglia are also vulnerable to environmental factors, such as infection and stress that may alter their phenotype and function. Viral infection activates microglia to produce inflammatory cytokines and anti-viral responses that protect the brain from damage. However, excessive or prolonged microglial activation impairs brain development and leads to long-term consequences such as autism spectrum disorder and schizophrenia spectrum disorder. Moreover, certain viruses may attack microglia and deploy them as “Trojan horses” to infiltrate the brain. In this brief review, we describe the function of microglia during brain development and examine their roles after infection through microglia-neural crosstalk. We also identify limitations for current studies and highlight future investigated questions.

## 1 Introduction

Microglia are resident mononuclear phagocytic cells in the central nervous system (CNS) that play a pivotal role in innate immune surveillance (Hickman et al., 2013). These cells arise exclusively from erythro-myeloid progenitors in the embryonic yolk sac (YS) and seed the brain early in development before birth (Palis and Yoder, 2001; Gomez Perdiguero et al., 2015). This early colonization of the developing brain by microglia precedes neurogenesis, neuronal wiring, and many other cellular processes, indicating their critical roles in mediating the normal occurrence of these events (Thion and Garel, 2017; Menassa and Gomez-Nicola, 2018). After birth, bone marrow-derived monocytes may migrate into the brain and integrate into the microglia population under certain circumstances such as brain injury or infection, making it difficult to distinguish from the resident microglia (Okonogi et al., 2014; Leovsky et al., 2015; Moravan et al., 2016; Jin et al., 2017; McKinsey et al., 2020).

As the first and major innate immune defender of the brain, microglia respond to invading pathogens (viral DNA/RNA) through “microglial sensome” to produce inflammatory cytokines and antiviral responses that may protect the brain from injury (Chhatbar and Prinz, 2021; Tran et al., 2022). However, sustained exposure of neurons to these inflammatory factors may result in neuronal dysfunction and damage that contribute to the pathogenesis of various neurological diseases (Filgueira et al., 2021). Particularly, microglial precursors have been implicated in transmitting certain neuroinvasive viruses to the embryonic brain and facilitating their dissemination within the CNS, leading to congenital infections and neurodevelopmental disorders (Dellacasa-Lindberg et al., 2011; Bielefeldt-Ohmann et al., 2012; Lum et al., 2017; Xu et al., 2021a).

**FIGURE 1:**
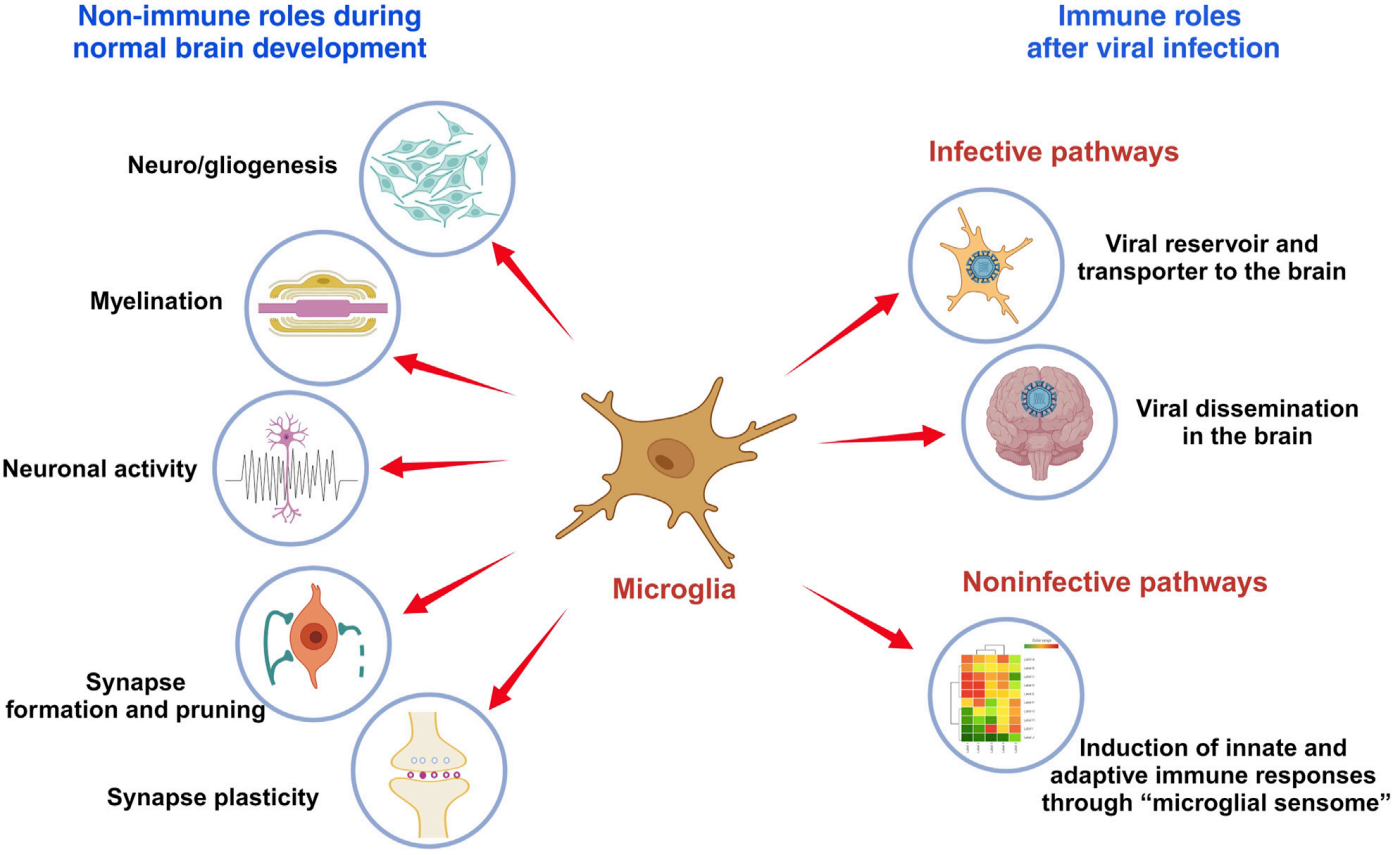
The role of microglia in brain development and immune responses. Microglia are immune cells in the brain that play critical roles in brain development and functionality, which include modulating neuro- and gliogenesis, myelination, neuronal activity, synapse formation and pruning, and synaptic plasticity. Viral infection activates microglia to produce inflammatory cytokines and anti-viral responses that may protect the brain from damage. But microglia also serve as viral reservoir and transporter for viral dissemination in the brain.

This minireview outlines the role of microglia in brain development and their function following infection (Figure 1). It also discusses limitations in current research and suggests areas for future investigation.

## 2 Role of microglia during brain development

### 2.1 The origin and characteristics of microglia

Microglia solely originate from erythro-myeloid progenitors located in the embryonic YS, an extra-embryonic membrane tissue that serves as the first site of hematopoiesis in both rodents and humans (Palis and Yoder, 2001; Colonna and Butovsky, 2017; Ornoy and Miller, 2023). In mice, the YS-derived microglial progenitors appear at embryonic day 7 (E7) to E8 and migrate to the brain as early as E9.5, following the establishment of blood vessels from the YS to the embryonic brain (McGrath et al., 2003; Ginhoux et al., 2010; Ginhoux and Prinz, 2015). In humans, microglia penetrate the cerebrum in a specific spatiotemporal pattern between the 4th and 24th gestational weeks (Monier et al., 2007; Menassa and Gomez-Nicola, 2018; Tan et al., 2020). Under steady-state conditions, YS-derived microglia are self-maintained throughout life by local proliferation. Furthermore, in the event of an injury to the adult brain, they proliferate in response to inflammation (Ginhoux et al., 2010; Ginhoux et al., 2013; Lenz and Nelson, 2018). Rodent YS-derived microglia are F4/80-bright and CD11b-dim characterized by flow cytometry (Schulz et al., 2012). However, under certain inflammatory conditions (e.g., viral encephalitis, irradiation, CNS cancer or neurodegenerative disorders), the recruitment of monocytes or other bone marrow-derived progenitors can supplement the microglial pool to some extent (Terry et al., 2012; Ginhoux et al., 2013; Sevenich, 2018). These hematopoietic stem cells (HSC)-derived macrophages are all F4/80-dim and CD11b-bright (Schulz et al., 2012).

### 2.2 Microglia control neurogenesis

Microglia colonize the developing brain early before neuro/gliogenesis, neuronal wiring, and many other cellular processes, suggesting their non-innate immune roles in supporting the brain development (Tay et al., 2017; Thion and Garel, 2017; Menassa and Gomez-Nicola, 2018; Mehl et al., 2022). They entry the brain rudiment in a stepwise manner and occupy specific layers of the prenatal cerebral cortex including the telencephalic proliferative zones, where they establish extensive contacts with neural precursor cells (NPCs) and developing cortical vessels (Monier et al., 2007; Matcovitch-Natan et al., 2016; Menassa and Gomez-Nicola, 2018; Tan et al., 2020; Penna et al., 2021a; Penna et al., 2021b). They help shape the newly formed CNS through regulating neurogenesis in multiple proliferative brain regions by phagocytic and secretory mechanisms (Lenz and Nelson, 2018; Mehl et al., 2022). In perinatal rodents and primates, studies have shown that microglial phagocytosis of NPCs controls NPC population density in the subventricular zone (SVZ) and inner SVZ of the developing cerebral cortex (Cunningham et al., 2013). The recruitment of microglia into the SVZ is influenced by C-X-C Motif Chemokine Ligand 12 (CXCL12) secreted from basal progenitors in the ventricular zone via microglial C-X-C motif chemokine receptor 4 (CXCR4) (Arnò et al., 2014). There, microglia extensively surveil the developing cortex via the CXCL12/CXCR4 system to promote the differentiation of NPCs at the mid-embryonic stage (Hattori and Miyata, 2018). Ablation of the essential microglial colony-stimulating factor-1 receptor (CSF-1R) signaling reduces the generation of cortical basal progenitor cells, further indicating the role of microglia in maintaining an appropriate progenitor cell pool (Arnò et al., 2014).

### 2.3 Microglia regulate gliogenesis

The transition from neurogenesis to gliogenesis is another important temporal event in the CNS development (Malatesta et al., 2000; Qian et al., 2000; Tong and Vidyadaran, 2016). This occurs in mice at around E16 until birth and in human from gestational weeks 21–26 (Malatesta et al., 2000; Qian et al., 2000; Estes and McAllister, 2016; Cadwell et al., 2019; Fu et al., 2021). At this stage, radial glial cells stop producing neurons and begin to generate astrocytes and later, oligodendrocytes (Qian et al., 2000; Tong and Vidyadaran, 2016). An *in vitro* study has shown that microglia induce the differentiation of astrocytes through the release of interleukin-6 (IL-6) and leukemia inhibitory factor (LIF) (Nakanishi et al., 2007). The depletion of resident embryonic microglia in PU.1 knock-out mice is associated with a decrease in the cortical precursor astrogenesis (Antony et al., 2011). Microglia also produce soluble factors, such as platelet-derived growth factor (PDGF), tumor necrosis factor alpha (TNF-α), IL-6, IL-1β, interferon gamma (IFN-γ) and insulin-like growth factor 1 (IGF1) to modulate the survival and maturation of oligodendrocytes (Nicholas et al., 2001; Shigemoto-Mogami et al., 2014; Hagemeyer et al., 2017). Specifically, a subset of CD11c^+^ microglia, found only in the developing white matter tracts of the early postnatal mouse brain, are important for oligodendrocyte development and myelinogenesis (Hagemeyer et al., 2017; Wlodarczyk et al., 2017). Combined, these findings highlight the multi-faceted ways in which microglia orchestrate neuro/gliogenesis through microglia-NPCs crosstalk to support a healthy CNS development.

### 2.4 Microglia shape neural circuits

Microglia help to shape neural circuits by eliminating dead cells, pruning synapses, and, in general, modulating emerging neuronal wiring (Wake et al., 2013; Squarzoni et al., 2014; Frost and Schafer, 2016; Lehrman et al., 2018). They remove excess neurons that fail to establish functional circuits during postnatal development (Bilimoria and Stevens, 2015; Colonna and Butovsky, 2017). Along this line, microglia seem to exert two distinct roles: firstly, they respond to programmed cell death by engulfing dead or dying neurons and related debris; secondly, they actively direct cell death through cues that are either soluble or contact-mediated (Bilimoria and Stevens, 2015). In terms of synaptic pruning, microglia trim neuronal synapses by engulfing dendritic spines that are not receiving inputs from synaptic contacts (Tremblay et al., 2011; Kettenmann et al., 2013). The complement system plays an indispensable role in microglia-mediated synaptic stripping (Stevens et al., 2007; Lenz and Nelson, 2018; Thion and Garel, 2018; Soteros and Sia, 2022). In the developing visual system of mice, the initiating protein in the classical complement cascade, complement component 1q (C1q), and the downstream complement protein C3 tag unwanted synapses for microglia recognition and removal via complement receptor 3 (CR3) (Stevens et al., 2007). Mice depleted of either C1q, C3, C4 or CR3 all exhibited defective synaptic pruning with redundant synapses (Stevens et al., 2007; Chu et al., 2010; Schafer et al., 2012; Sekar et al., 2016). This microglia-mediated and complement-dependent synaptic refinement takes place in different regions of the developing CNS, and alterations in this process cause behavior changes such as seizures and schizophrenia in mice (Chu et al., 2010; Sekar et al., 2016). Human brain development follows a protracted time course over which synapse elimination occurs (Huttenlocher, 1979; Petanjek et al., 2011). This creates an extended window of susceptibility for harmful environment-gene interactions, contributing as a significant player in the pathogenesis of neurodevelopmental disorders (Magdalon et al., 2020; Soteros and Sia, 2022).

### 2.5 Microglia affect neuronal activity

Besides sensing the neuronal activity through their recruitment to synapses and subsequent engulfment, microglia may also regulate the synaptic activity via non-phagocytic interactions (Wong and Favuzzi, 2023). For example, microglia sense extracellular ATPs released by the active neurons and mobilize to the active synapses (Badimon et al., 2020). The microglial ectoenzyme CD39 hydrolyzes ATP to AMP, which is converted into adenosine by CD73, an enzyme expressed on microglia and other brain cells (Badimon et al., 2020). Adenosine then suppresses presynaptic neuronal activity via the neuronal adenosine receptor A1R (Badimon et al., 2020). This microglia-driven negative feedback mechanism functions similarly to the inhibitory neurons and plays a critical role in preventing the brain from becoming overactive in both healthy and diseased states (Badimon et al., 2020). Microglia may also shape the neuronal properties and connectivity by remodeling the extracellular matrix (ECM) (Nguyen et al., 2020; Wong and Favuzzi, 2023). A recent study showed that microglia have the ability to remodel the ECM in the context of experience-dependent plasticity (Nguyen et al., 2020). Neuron-derived IL-33 guides microglial clearance of the ECM, leading to alterations in synapse plasticity and memory consolidation (Nguyen et al., 2020).

Collectively, microglia affect the brain wiring by controlling the neuronal numbers and migration, synapse formation and plasticity, neuronal activity, as well as ECM remodeling during development and throughout life (Wong and Favuzzi, 2023).

## 3 Role of microglia during brain development after infection

Given the versatile functions of microglia as CNS architects, any insults that impair microglia during brain development can result in defective elimination of cellular debris and compromised neural circuitry. This perturbation of microglial homeostasis can directly contribute to the onset of several neurological disorders that arise from the disrupted neurodevelopment either early in life or later in adulthood. Infection, stress, and dietary intake are well-characterized environmental stimuli associated with microglia-mediated aberrant neurogenesis and synaptic dysfunction (Marques et al., 2013; Marques et al., 2015; Paolicelli and Ferretti, 2017; Wen et al., 2017; Xu et al., 2021a). Particularly, microglia may progress to a more competent immune-sensing phenotype during the early embryonic development, rendering the fetal brain more susceptible to environmental insults throughout the early pregnancy (Knuesel et al., 2014; Kracht et al., 2020).

### 3.1 Microglia in the brain after viral invasion: the infective pathway

A broad range of viruses may cause acute or delayed neurological manifestations in both animals and human. Notably, neurotropic virus infection along with the subsequent immune responses may cause permanent disturbance of the functional structure in the brain, leading to significant clinical phenotypes (Bielefeldt-Ohmann et al., 2012; Wang et al., 2018; Lannes et al., 2019; Stonedahl et al., 2020; Xu et al., 2021a; Chhatbar and Prinz, 2021). These include members of the *Flaviviridae* virus family, such as Zika virus (ZIKV), bovine viral diarrhea virus (BVDV) and Japanese encephalitis virus (JEV), which are enveloped, positive sense, single stranded RNA (ssRNA) viruses with neuroinvasive features (Neal, 2014). Studies have shown that microglia can serve as a viral reservoir for the transmission and dissemination of ZIKV, BVDV and JEV in the CNS due to the high permissiveness of microglia to these viruses (Thongtan et al., 2010; Bielefeldt-Ohmann et al., 2012; Thongtan et al., 2012; Meertens et al., 2017; Muffat et al., 2018; Lannes et al., 2019; Malmlov et al., 2019; Xu et al., 2021a). During development, YS-derived embryonic microglia enter and colonize the fetal brain even before the appearance of the complete brain vasculature, the blood-brain barrier and neurogenesis (Tan et al., 2020). A viral attack on the mother during early pregnancy may induce substantial neurodevelopmental disorders in the embryo and adult offspring (Thion et al., 2018). As observed in the ZIKV-infected mothers in both animals and humans, infections during early pregnancy are associated with a higher risk of fetal demise and brain malformation than infections occurred later (Brasil et al., 2016; Kleber de Oliveira et al., 2016; Miner et al., 2016; Yockey et al., 2016; Coelho and Crovella, 2017; Valentine et al., 2018; Xu et al., 2021a). *In vitro* studies have found that YS-derived microglia progenitors are susceptible to ZIKV, and can produce and release progeny viruses into the surroundings (Xu et al., 2021a). Infected microglia progenitors may act as “Trojan horses” to transport ZIKV from the mother to the fetal brain during their migration (Xu et al., 2021a). ZIKV directly targets NPCs in the embryonic brain and over-activates innate immune responses, leading to disrupted cell cycling, reduced neurogenesis and impaired synaptogenesis (Hammack et al., 2019; Rosa-Fernandes et al., 2019; Xu et al., 2021b). BVDV, another neuroinvasive virus that can spread from mother to fetal brain, may also deploy microglial precursors as transporters (Bielefeldt-Ohmann et al., 2012). Meanwhile, sustained and strong IFN and inflammatory responses were observed in the infected fetal brain, which may add to insults to the brain development (Bielefeldt-Ohmann et al., 2012). Microglia can also transmit viral infectivity to surrounding cells in a cell-to-cell contact-dependent manner through the CX_3_CR1-CX_3_CL1 axis, as shown in the JEV infection (Lannes et al., 2019). The crosstalk between the CX_3_CR1-expressing microglia and CX_3_CL1-expressing neurons may significantly contribute to the dissemination of the virus in the brain and the persistence of JEV neuropathogenesis over time (Lannes et al., 2019).

### 3.2 Microglia in the brain after viral invasion: the non-infective pathway

#### 3.2.1 Microglia sense viral attacks through the “microglial sensome” to induce immune responses

**FIGURE 2:**
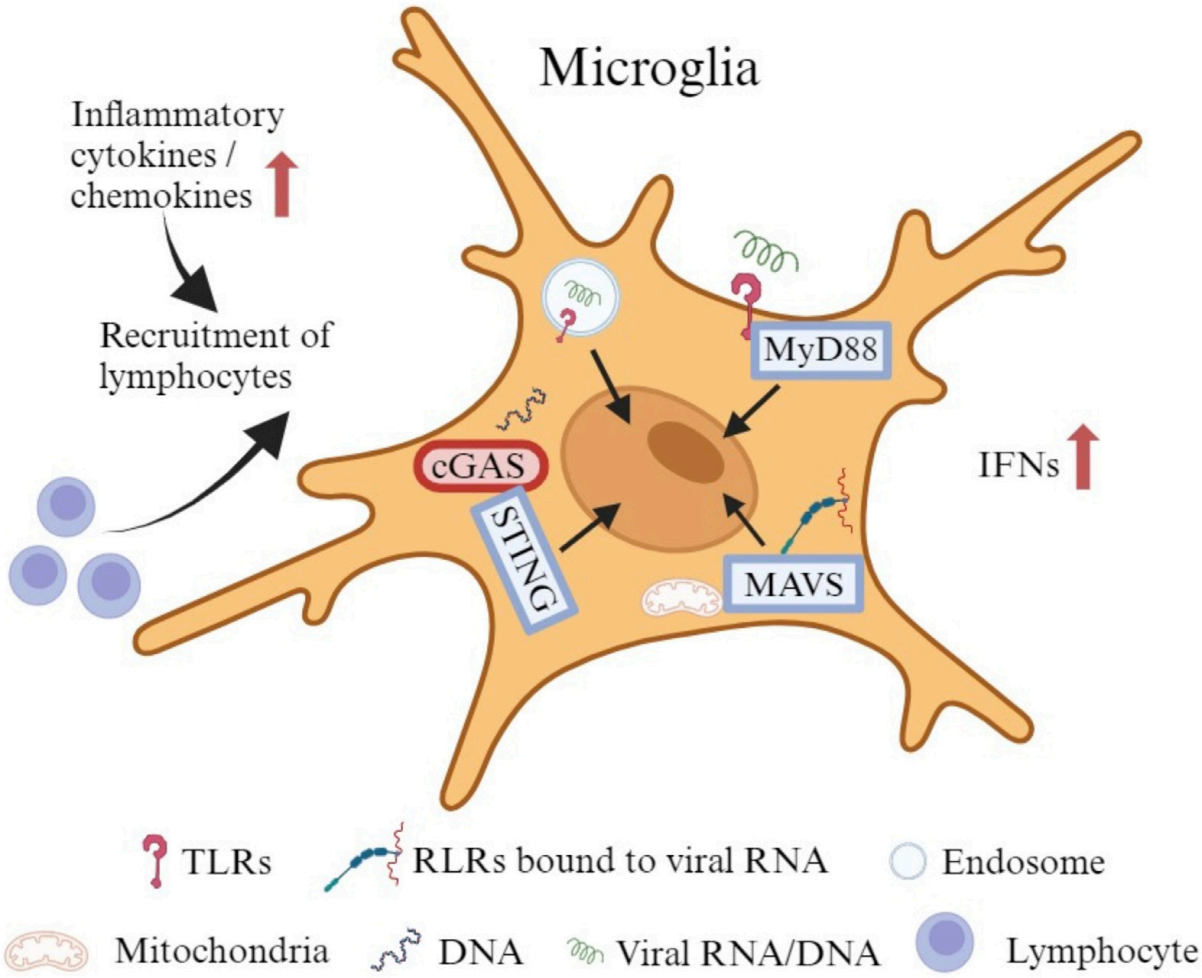
The immune responses of microglia to viral infection. Microglia express all TLRs to sense viral RNA/DNA in the environment and endosomal compartments. Certain TLRs utilize MyD88 as an adaptive molecule to trigger a downstream signaling cascade. Microglia also express RLRs such as RIG-I and MDA5 to identify viral RNA in the cytoplasm. With the adapter molecule MAVS, RIG-I/MDA5 leads to the transcriptional activation of IFNs and other pro-inflammatory cytokine genes. In addition, microglia exhibit robust expression of cGAS and the downstream adaptor STING at rest and upon activation. cGAS recognizes both exogenous and endogenous DNA to initiate potent type-I IFN and pro-inflammatory responses through STING. The production of cytokines and chemokines may attract lymphocytes to promote an adaptive immune response.

As the primary innate immune cells of the CNS, microglia also actively and efficiently function in the brain through noninfective pathways after viral invasion (Thongtan et al., 2012). They can stimulate both innate and adaptive immune responses to protect the host. However, under some circumstances, they trigger an uncontrolled immune response with secretion of chemokines and cytokines to recruit peripheral immune cells that cause an inflammatory cascade (Figure 2), which can also disturb the normal brain development (Chhatbar and Prinz, 2021). Microglia express a wide range of proteins that vigilantly sense the invading microbes, dying cells, as well as exogenous and endogenous ligands, which are described as the “microglial sensome” (12). Both extracellular pattern recognition receptors (PRRs), such as Toll-like receptors (TLRs), and intracellular PRRs, like retinoic acid inducible gene (RIG)-I-like receptors (RLRs), recognize components of exogenous pathogens termed pathogen-associated molecular patterns (PAMPs) and endogenous molecules released from damaged cells named damage-associated molecular patterns (DAMPs) (Kwon and Koh, 2020; Chhatbar and Prinz, 2021). Microglia express all TLRs to sense viral RNA/DNA in the environment and endosomal compartments (Olson and Miller, 2004; Xagorari and Chlichlia, 2008). TLRs use myeloid differentiation primary response 88 (MyD88) as an adaptive molecule, with the exception of TLR3 and TLR4 that utilize the TIR-domain-containing adaptor-inducing interferon-β (TRIF)-dependent pathway to activate a downstream signaling cascade (Kawai and Akira, 2007; Kawai and Akira, 2010; Takeuchi and Akira, 2010; Shalaby et al., 2017). Microglia also express RLRs such as RIG-I and melanoma differentiation-associated protein 5 (MDA5) to detect the viral RNA in the cytoplasm (Chhatbar and Prinz, 2021). With the adapter molecule mitochondrial antiviral-signaling protein (MAVS), RIG-I/MDA5 leads to the transcriptional activation of IFN and pro-inflammatory cytokine genes (Onomoto et al., 2021). In addition, microglia show a robust expression of cyclic GMP-AMP synthase (cGAS) and the downstream adaptor stimulator of interferon genes (STING) both at rest and following activation (Jeffries and Marriott, 2017). cGAS recognize both exogenous DNA introduced into the cells by invading pathogens and endogenous DNA released from the nucleus and mitochondria, to initiate potent type-I IFN and pro-inflammatory responses through STING (Reinert et al., 2016; Fryer et al., 2021).

#### 3.2.2 Uncontrolled immune responses induced by microglia may disrupt normal brain development

Studies have shown that ZIKV-infected microglia express high levels of type I (IFN-α and IFN-β) and type II (IFN-γ) IFN, as well as neurotoxic factors (IL-6, IL-12, TNF-α, IL-1β, CCL2, iNOS and NO) that aggravate the inflammatory injury. Such uncontrolled immune responses may result in impaired neurogenesis, especially in the cortex, and lead to a spectrum of neuropathological changes, including microcephaly, seizures, and vision or hearing problems in offspring (Lum et al., 2017; Diop et al., 2018; Mesci et al., 2018; Wang et al., 2018; Elgueta et al., 2022; Komarasamy et al., 2022). Histopathological assessments of the brains damaged by congenital ZIKV infection have shown scattered microglial nodules, microcalcifications and necrosis (Martines et al., 2016). Activated microglia with a robust, unrestricted induction of IFN response have also been detected in the fetal brain from BVDV-challenged dams (Bielefeldt-Ohmann et al., 2012). Persistent immune activation may be involved in the formation of vascular lesions and microcysts in the developing brain (Bielefeldt-Ohmann et al., 2012). Cytomegalovirus (CMV), a double-stranded DNA virus, is one of the well-known TORCH viruses that may cause serious neurodevelopmental sequelae, including microcephaly, seizures, developmental delay, and sensorineural hearing loss (Tsutsui, 2009; Gowda et al., 2021). Microglia are highly permissive to CMV and can support productive CMV replication (Schut et al., 1994). An early activation of microglia has been identified at the site of CMV-infected fetal brains, with an infiltration of peripheral immune cells, as well as an increased production of cytokines and chemokines (Cheeran et al., 2001; Cloarec et al., 2016). Early microglial responses fight well against CMV infection, whereas uncontrolled immune activation may have deleterious effects through interactions with key neurodevelopmental processes (Cloarec et al., 2016; Cloarec et al., 2018). As shown in the histopathological studies from autopsied fetuses with congenital CMV infection, microglial nodules containing CMV-positive cells surrounded by activated CD8^+^ T-cells were uniformly distributed in damaged brains (Teissier et al., 2014; Piccirilli et al., 2023). Besides aforementioned severe neurological manifestations, microglia are also involved in some mild, prolonged but irreversible changes that play critical roles in slow progressive neurodevelopmental disorders such as autism spectrum disorders (ASD) (Kaur et al., 2017; Slawinski et al., 2018). Congenital CMV infection has been reported to be linked with the onset of ASD, in which microglial dysfunction affects the dendritic spine pruning, causing abnormality in neuronal connectivity and brain development (Slawinski et al., 2018; Soteros and Sia, 2022).

Recently, two neonates born to mothers with severe acute respiratory syndrome coronavirus disease 2 (SARS-CoV-2) infection have been reported to show seizures, microcephaly, and over time, considerable developmental delay (Benny et al., 2023). Neurological, cognitive, and psychiatric sequelae have also been identified in patients months after the SARS-CoV-2 infection (Jeong et al., 2022; Pandharipande et al., 2023). In rodent and primate studies, maternal infection with influenza during pregnancy has also been associated with a higher risk for schizophrenia and other neurodevelopmental disorders (Fatemi et al., 1999; Fatemi et al., 2008; Short et al., 2010). Whether SARS-CoV-2 is neurotropic remains a subject of debate, and only a few influenza strains possess the ability for neurotropism (Aronsson et al., 2002; Gu et al., 2007; Aschman et al., 2022; Proust et al., 2023). However, microglia are known to express entry factors for SARS-CoV-2, including angiotensin converting enzyme 2 (ACE2) and transmembrane protease serine subtype 2 (TMPRSS2) (Qiao et al., 2020; Singh et al., 2020; Jeong et al., 2022; Proust et al., 2023). Using human ACE2 knock-in mice model, viral infection has been detected in the brains of pregnant mice after an intranasal inoculation with SARS-CoV-2, as well as in the brains of their fetuses (McMahon et al., 2023). Moreover, histological studies have revealed that SARS-CoV-2 localizes within the microglia of fetal brains, and a reactive morphology is observed long after the virus has been cleared (McMahon et al., 2023). Activation of microglia could trigger neuroinflammatory processes, including microgliosis, immune cell accumulation, and cell death, thereby, serving as a key mechanism contributing to neuroinflammation and neurological complications (Jeong et al., 2022). Unlike with SARS-CoV-2, microglia play a more indirect role in the fetal brain development following maternal infection with influenza, primarily through maternal immune activation (MIA) (Otero and Antonson, 2022). Viral pathogens, which include non-vertically transmitted influenza viruses, remain the predominant environmental cause of MIA (Massrali et al., 2022). During MIA, pro-inflammatory cytokines, specifically IL-6 and IL-17A, are generated (Elgueta et al., 2022; Otero and Antonson, 2022). They cross the placenta to prime fetal brain microglia toward a highly proinflammatory phenotype, leading to the occurrence of neurodevelopmental abnormalities in the fetal brain (Elgueta et al., 2022; Otero and Antonson, 2022).

In summary, following an infection, microglia may adversely impact neurodevelopment either through the infective pathway that microglia act as a viral reservoir to transport and disseminate the virus throughout the brain; or via the noninfective pathway that provoke an unrestricted immune response with a cytokine storm, compromising the normal brain development (Table 1).

**TABLE 1:** Impact of common viruses on microglia and immune reactions.

Viruses	Impact of microglia	Immune responses
Z1KV	Viral reservoir for the transmission and dissemination	Innate and adaptive immune responses
	Production of type I and type IIIFNs, as well as neurotoxic factors	
BVDV	Viral reservoir for the transmission and dissemination	Type 1 11Th response
	Intense type I IFN-stimulated protein ISG 15 expression	
JEV	Viral reservoir for the transmission and dissemination	Innate and adaptive immune responses
	Production of inflammatory mediators	
CMV	Viral reservoir for the transmission and dissemination	Innate and adaptive immune responses
	Increased cytokines and chemokines production	
SARS-CoV-2	Viral reservoir?	Innate and adaptive immune responses
	Proinflammatory activation and M1 phenotype polarization	
Influenza	Viral reservoir?	Innate and adaptive immune responses
	Increased expression of cytokines and chemokines	

## 4 Discussion, current research gaps and future prospects

Despite tremendous advances that have been made in clarifying the role of microglia in sculpting the developing brain and how microglia exert their function following infection during early brain development, many important mechanistic details are still not clear. Below, we present some of the major unanswered questions in the field.

Firstly, microglia serve as a key player in building the normal brain histoarchitecture including phagocytosis of dead cells, pruning of redundant synapses, and modulate the strength of synaptic transmissions to remold neural circuits. The development of microglia and CNS is an intimate journey (Hammond et al., 2018; Thion et al., 2018). Emerging literature is unveiling a stepwise developmental program that shapes microglia from progenitors in the YS to mature cells in developed brain at different stages and how it synchronizes with the formation of the brain (Matcovitch-Natan et al., 2016). Most of the data are obtained from the *in vivo* animal models, 2D cell cultures, and 3D microglia-containing human brain organoids (Zhang et al., 2023). However, the human and rodent brains have different microglial signatures and regional distributions. How these anatomic/molecular disparities contribute to functional distinction remains unclear. 2D cell cultures could not capture the *in vivo* signatures of microglia. 3D microglia-containing human brain organoids at the current stage cannot recapitulate the real human brain microenvironment in all respects (Zhang et al., 2023). To better understand the roles of microglia in brain development under both healthy and diseased conditions, a robust microglial model that mimics the *in vivo* human brain is required.

Secondly, although neurons are thought to be the primary target of neurotropic viruses in the brain, it is the activation of microglia that causes an exaggerated inflammatory response and subsequent neuronal damages. This is particularly concerning since microglia are the main brain defender responsible for safeguarding neurons from infections. The question then arises: why this neuroprotective mechanism leads to apparently increased damage to neurons? Microglia become activated both directly as a consequence of the infection within themselves, as well as indirectly as a result of the neuronal damage. Moreover, infected and activated microglia release chemotactic mediators such as CCL2 and CXCL12 that recruit additional monocyte populations from the periphery to invade the brain (Yadav and Collman, 2009; Cloarec et al., 2016; Kaur et al., 2017). These invasive, activated and partially infected monocytes release viral proteins, neurotoxic products and inflammatory mediators that act on bystander cells and in turn activate them, thereby amplifying the inflammatory cascade (Yadav and Collman, 2009). However, the term “activation” does not sufficiently capture the variety of microglial responses to the environmental changes or the diversity of their functional statuses. A recent study suggests that Pellino-1, an E3 ubiquitin ligase expressed in microglia/macrophages, induces fetal demise and intrauterine growth restriction by promoting ZIKV infection and inflammatory cytokine production (Luo et al., 2020). However, the role of Pellino-1 in ZIKV-associated brain malformation is unknown. Elucidating the transcriptional and epigenomic alternation in microglial activation may pave the way not only for a better understanding of the dynamic profile of microglia, but also for exploring appropriate therapeutic targets to mitigate the neurotoxic effects (Kaur et al., 2017).

Thirdly, as mentioned, the exacerbated inflammatory response primed by microglia can detrimentally affect normal neurodevelopment. This raises the question: are there any tools to dampen the proinflammatory response of microglia while preserve the neuroprotective function? Recent studies have shown numerous compounds that can intervene in the inflammatory process to increase the neuroprotective activity as well as reduce the pro-inflammatory cytokine production in an experimental JVE murine model (Swarup et al., 2007; Thongtan et al., 2012). Administration of tetracycline derivative doxycycline to pregnant dams throughout pregnancy improves the microglial phenotype of the offspring, which is related with improved neurodevelopmental outcomes after CMV infection in rats (Cloarec et al., 2018). Importantly, human microglia are highly heterogeneous during early embryonic development and maturation by mid-gestation, which makes the developing brain extremely vulnerable to any maternal immune dysregulation or/and environmental perturbation (Kracht et al., 2020). Therefore, therapeutic interventions targeting microglia should work friendly with both the pregnant woman and the developing fetus. By better deciphering the potential molecular/cellular mechanisms and pathways of neurological manifestations, novel strategies for preventing and treating neurodevelopmental and neurodegenerative diseases can be developed.

## Abbreviations

ASD, Autism spectrum disorders; BVDV, Bovine viral diarrhea virus; cGAS, Cyclic GMP-AMP synthase; CMV, Cytomegalovirus; CNS, Central nervous system; CXCL, C-X-C Motif chemokine ligand; ECM, Extracellular matrix; GW, Gestational week; JEV, Japanese encephalitis virus; IFN, Interferon; IL, Interleukin; CCL2, Monocyte chemoattractant protein-1; MDA5, Melanoma differentiation-associated protein 5; NPC, Neural precursor cell; RIG, Retinoic acid inducible gene; PRR, Pattern recognition receptor; STING, Stimulator of interferon genes; SVZ, Subventricular zone; TLR, Toll-like receptors; TRIF, TIR-domain-containing adaptor-inducing interferon-β; YS, Yolk sac; ZIKV, Zika virus.
